# Supplementary figures and images for: Hypergraph modeling of complex interactions: Applications from human musculoskeletal structures to complex system dynamics
Source: PLoS One. 2024 Nov 12;19(11):e0310189. doi: 10.1371/journal.pone.0310189 (PMC11556689; doi:10.1371/journal.pone.0310189)

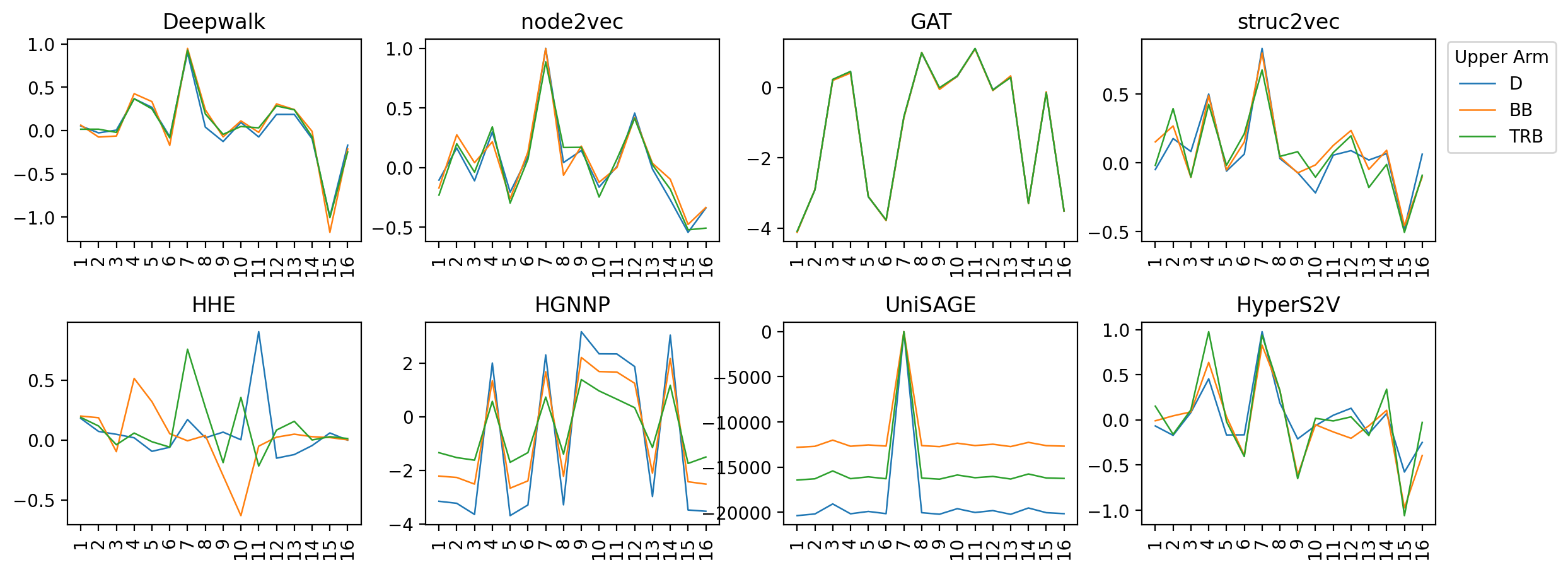

Supplement: S1 Fig — (16 dimensions). (PNG) [file pone.0310189.s001.png]

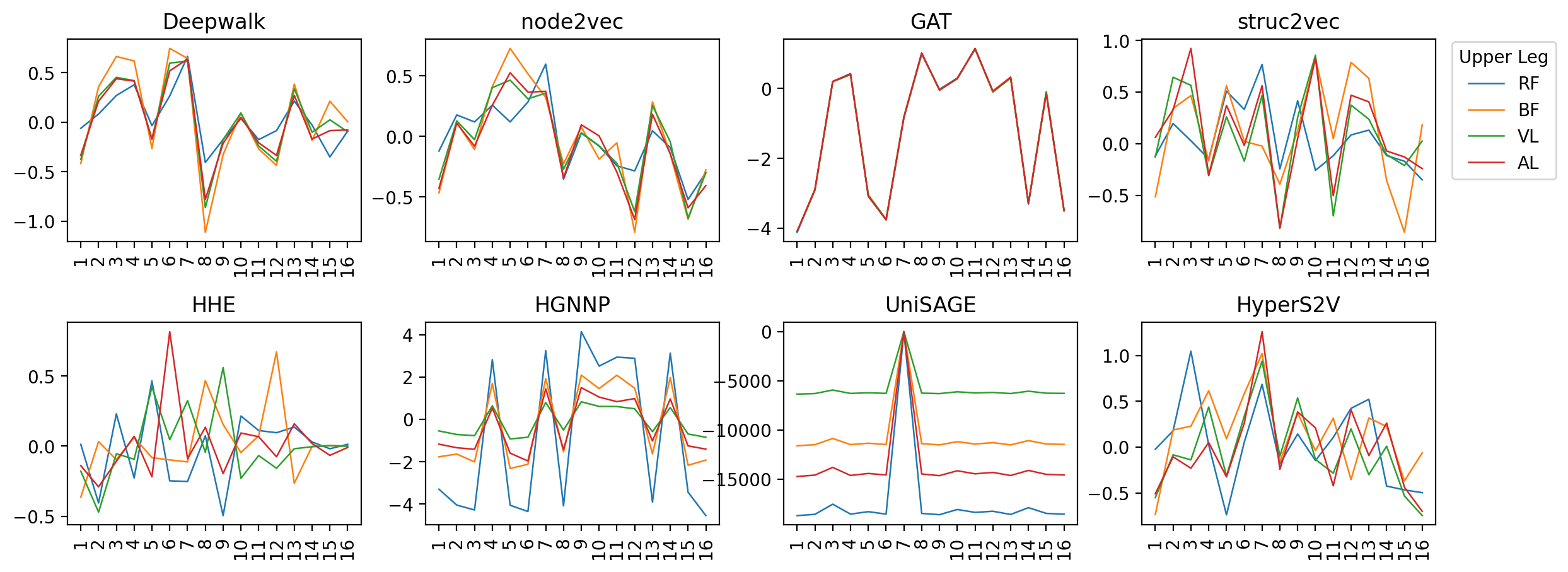

Supplement: S2 Fig — (16 dimensions). (PNG) [file pone.0310189.s002.png]

HyperS2V result on 18 muscle embeddings (Body\_parts, PCA)

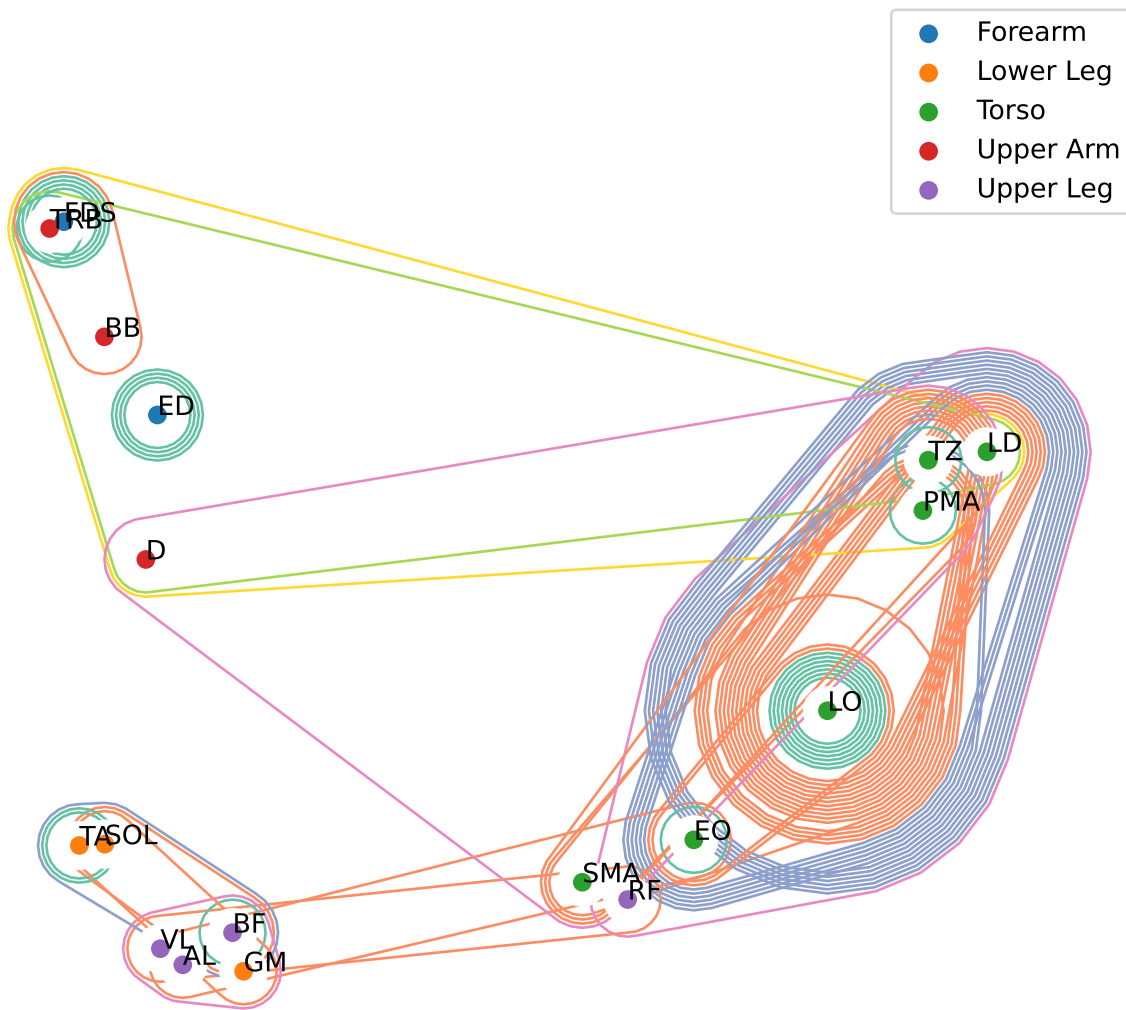

Supplement: S3 Fig — (PDF) [file pone.0310189.s003.pdf]

struc2vec result on 18 muscle embeddings (Body\_parts, PCA)

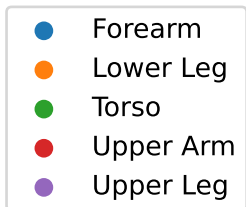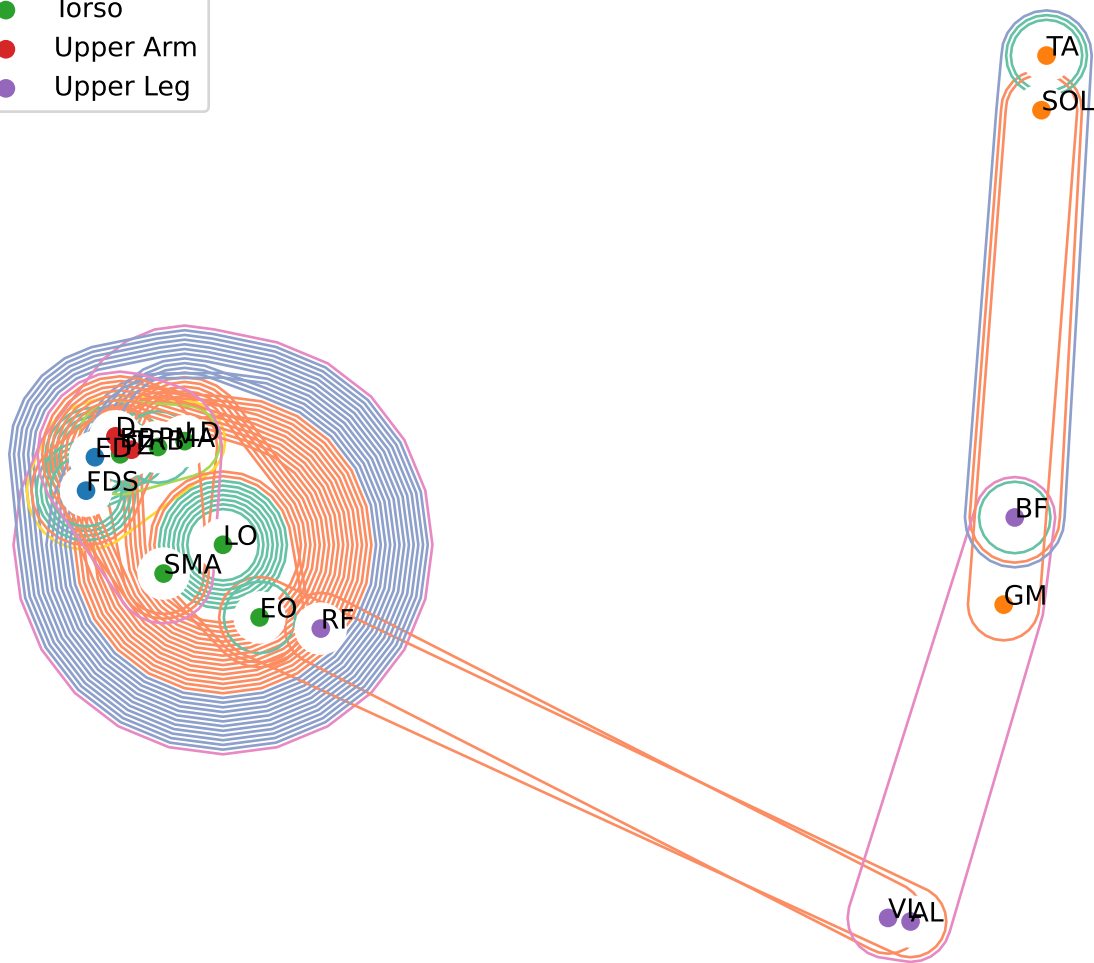

Supplement: S4 Fig — (PDF) [file pone.0310189.s004.pdf]

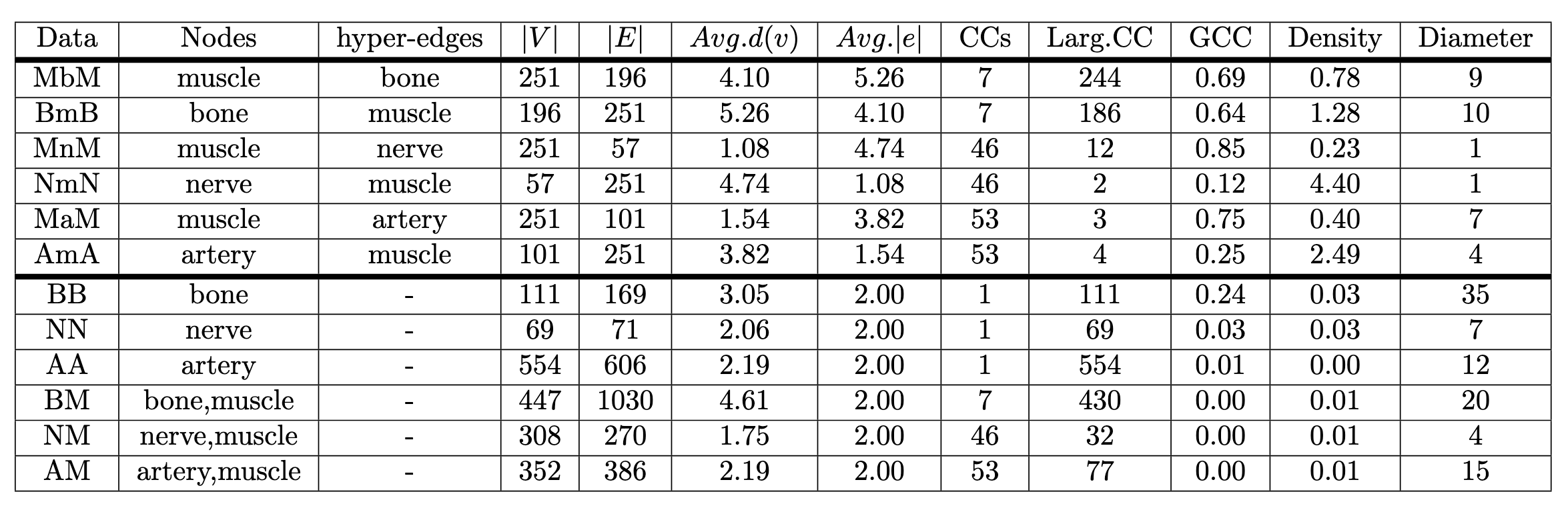

Supplement: S1 Table — (PNG) [file pone.0310189.s005.png]
